# The Atherosclerosis Profile by Coronary Computed Tomography Angiography (CTA) in Symptomatic Patients with Coronary Artery Calcium Score Zero

**DOI:** 10.3390/diagnostics12092042

**Published:** 2022-08-24

**Authors:** Gudrun Feuchtner, Christoph Beyer, Fabian Barbieri, Philipp Spitaler, Wolfgang Dichtl, Guy Friedrich, Gerlig Widmann, Fabian Plank

**Affiliations:** 1 Department of Radiology, Innsbruck Medical University, 6020 Innsbruck, Austria; 2 Department of Cardiology, Charité—Universitätsmedizin Berlin, Hindenburgdamm 30, 12203 Berlin, Germany; 3 Department of Internal Medicine III, Cardiology, Innsbruck Medical University, Cardiology, 6020 Innsbruck, Austria

**Keywords:** coronary computed tomography angiography (CTA), coronary artery disease, coronary artery calcium score (CACS), high-risk plaque (HRP), atherosclerosis, non-obstructive coronary artery disease

## Abstract

(1) Background: Whether it is safe to exclude coronary artery disease (CAD) in symptomatic patients with coronary artery calcium score (CACS 0), is an open debate. To compare coronary CTA including high-risk plaque (HRP) features in symptomatic patients with CACS 0 (2) Methods: 1709 symptomatic patients (age, mean 57.5 ± 16 years, 39.6% females) referred to coronary CTA for clinical indications were included. CACS, coronary stenosis (CADRADS) severity and HRP features (low-attenuation-plaque, spotty calcification, positive remodeling, NRS) were recorded. (3) Results: Of 1709 patients, 665 with CACS 0 were finally included. 562 (84.5%) had no CAD by CTA while 103 of 665 (15.4%) had CAD. Stenosis was minimal <25% in 79, mild <50% in 20, moderate in 1 and severe >70% in 3 patients. The rate of obstructive CAD was low with 4/665 (0.61%). The majority of patients had non-obstructive CAD (<50% stenosis) (99/103; 96.1%). A high proportion of patients with non-obstructive CAD had at least one HRP (52/103; 50.4%) per patient. (4) Conclusions: The rate of obstructive CAD is very low in symptomatic patients with CACS 0, and non-obstructive CAD domineering. CACS 0 does not rule out non-obstructive CAD and misses patients in which primary preventive measures are indicated. More than half of patients with non-obstructive CAD had high-risk plaque, highlighting the importance of quantitative plaque analysis.

## 1. Introduction

Coronary computed tomography angiography (CTA) is the modality of choice in patients with chronic coronary syndromes [1] and stable chest pain [2,3]. In contrast, coronary artery calcium scoring (CACS) is a valuable tool for cardiovascular risk stratification [4]. A CACS of 0 has a high prognostic value to rule out CAD in low-to-intermediate risk asymptomatic individuals. However, whether CACS of 0 is as reliable in symptomatic patients, including those at a higher probability of ischemia based on prior testing, is an open matter of debate. Mortensen et al. [5] has recently shown that the prevalence of obstructive CAD is low (3–7%) while non-obstructive disease prevalence is markedly higher, and strongly depends on age. The majority (58%) of younger patients below 40 years of age with obstructive CAD had CACS of 0, raising considerable concern about safety. However, the prevalence declined linearly with age to only 5% in those above 70 years. [5] Importantly, CACS 0 was found to be safe with regards to fatal endpoints. However, Mortensen et al. [5] did not include an analysis of “high-risk” plaque (HRP). Therefore, we aim to report the atherosclerosis profile (stenosis severity) including “high-risk-plaque” criteria as novel feature, in consecutive symptomatic patients referred to coronary CTA with novel (2nd and 3rd generation) dual source computed tomography (CT). High-risk plaques (HRP) are strong independent prognosticators for adverse cardiovascular (CV) outcomes [6,7,8,9,10,11] and ischemia [12], hence identification of such patients is of high clinical relevance for defining primary prevention recommendations.

## 2. Materials and Methods

Patients who underwent ECG-gated coronary CTA for clinical indications were included into this retrospective cohort study. Institutional review board (IRB) approval was obtained.

**Inclusion criteria were**: Patients with “low-to-intermediate probability of coronary artery disease”—either due to atypical or typical chest pain and/or other prior test findings suspicious of ischemia, such as resting ECG, treadmill ECG test, echocardiography, 24 h Holter ECG monitoring or myocardial perfusion imaging (SPECT), who met a clinical indication for referral to coronary CTA [1].

**Excluded** were patients

- With known CAD such as those after percutaneous coronary intervention (PCI) or coronary artery bypass grafting (CAGB).
- Referred for planning of minimal invasive valve intervention (e.g., transcatheter aortic valve intervention (TAVI) or mitral valve intervention or other structural heart disease indication such as left atrial catheter ablation).
- Referred for congenital heart disease (CHD) evaluation or other indications such as cardiac valves or masses.

### 2.1. Cardiac Computed Tomography

#### 2.1.1. Coronary Artery Calcium Score (CACS)

A non-contrast ECG-gated CT scan with standardized scan parameters (detector collimation 64 × 1.5 mm, 120 kV; image reconstruction 3 mm slice width, increment 1.5), and prospective ECG-triggering was performed. The Agatston Score (AU) was calculated.

#### 2.1.2. Coronary CTA

Coronary CTA was performed was performed by using 128-slice dual-source CT (*Definition FLASH or DRIVE, Siemens Healthineers, Erlangen, Germany*) with a detector collimation of 2 × 64 × 0.6 mm and a rotation time of 0.28 s. Scans were triggered into arterial phase using bolus tracking (threshold of 100 HU, ascending aorta) and by injecting an intravenous iodine contrast agent (*Iopromide, Ultravist 370*™, Bayer Healthcare, Berlin, Germany). Prospective ECG-triggering was applied in patients with regular heart rates <65 bpm (70% of RR-interval) and retrospective ECG-gating in those with >65 bpm and irregular rhythm. Axial thin slice images were reconstructed with 0.75 mm slice width (increment, 0.4) and transferred to a 3D-postprocessing software (*SyngoVIA, Siemens Healthineers, Erlangen, Germany*).

*Coronary CTA Image Analysis.* Coronary arteries were evaluated using curved multiplanar reformations (MPR) for atherosclerotic plaque and coronary stenosis. Coronary stenosis was graded according to CAD-RADS as minimal (1) <25%, mild (2) 25–49.9%, moderate (3) 50–69.9%, severe (4) ≥70–99% and (5) occluded 100% [13], subjectively, by an experienced observer with 10 years of cardiac CT experience. Subjective grading was assisted by quantitative stenosis measurement (area and diameter stenosis) in selected patients, in whom the lesion was accurate traceable and not severely calcified.

High-risk plaques (HRP) were defined according to standardized criteria (low attenuation plaque <60 HU, positive remodeling, spotty calcification and Napkin-ring sign) [6,7,8,9,10,11].

- Low attenuation plaque (LAP) was defined as hypoattenuating lesion with <150 Hounsfield units (HU). CT-density was screened with the “pixel lens” and the lowest HU recorded. Then, a region-of-interest (ROI) of approximately 2 mm^2^ size was placed at the region of lowest density and drawn as large as possible, while sparing areas affected by artifacts or adjacent to calcifications and the CT-attenuation (HU) quantified. If a patient had multiple lesions, the one with the lowest HU was selected. Low-attenuation plaque (LAP) was defined as LAP <30 HU (lipid-rich necrotic core) and <60 HU.
- Napkin-ring sign was defined as an outer high-density rim with an inner hypodense area.
- Spotty calcification was defined as a calcification of less than 3 mm size.
- The remodeling index was calculated as the ratio of the maximal cross-sectional lumen of the plaque diameter and its closest proximal (or distal, e.g., in case of ostial lesions) normal reference vessel lumen diameter. Positive remodeling was defined as remodeling index >1.1.

A high-risk plaque (HRP) was defined if a minimum of 2 out of 4 criteria were present (CAD-RADS/V) [13].

Coronary CTA image analysis was performed by two independent observers (one observer with SCCT level II training, and one observer with SCCT level III training and 10 years of cardiac CT experience). Consensus reading was obtained.

## 3. Results

Of 2128 patients referred to coronary CTA, 1709 patients (age, mean 57.5 years ± 16, 39.6% females) who met the inclusion criteria, were enrolled. Table 1 shows the population profile and Table 2 the cardiovascular risk factors (CVRF) in patients with CACS 0 and CACS positives (>1AU). Arterial hypertension and diabetes were more prevalent in CACS positives. Age, gender and other CVRF were not different.

Out of 1709, 666 (39%) had CACS 0. One patient with CACS 0 had non-diagnostic CTA image quality (CADRADS/N) due to artifacts (motion artifacts and high image noise due to obesity) and was excluded. Finally, 665 patients were included for CTA image analysis (Table 3).

562 of 665 (84.5%) had no CAD by CTA while 103 of 665 (15.4%) had CAD. Stenosis was minimal <25% (CADRADS 1) in 79 patients, mild <50% (CADRADS 2) in 20, moderate 50–70% (CADRADS 3) in 1 and severe >70% (CADRADS 4) in 3. (Table 2). In total, the rate of obstructive CAD was low (4/103, 3.9%) in those who had CAD, and 4/665 (0.61%) in all those who had CACS 0. The rate of non-obstructive CAD (<50% stenosis) was higher with 99/103 (96.1%) and 99/655 (15.1%) of all those with CACS 0.

**Figure 1:**
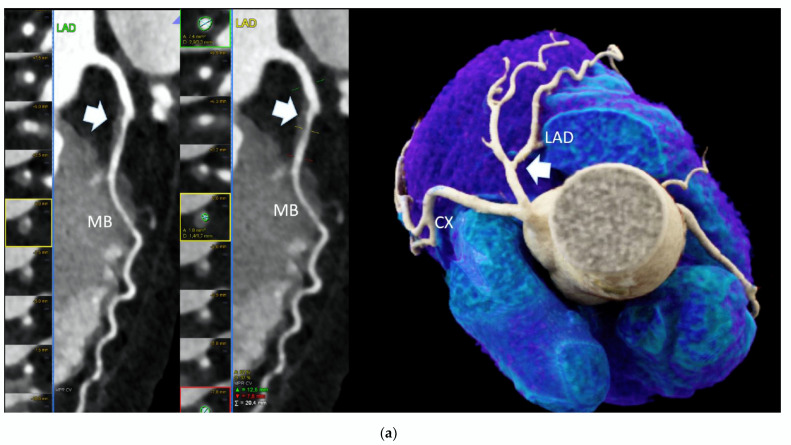

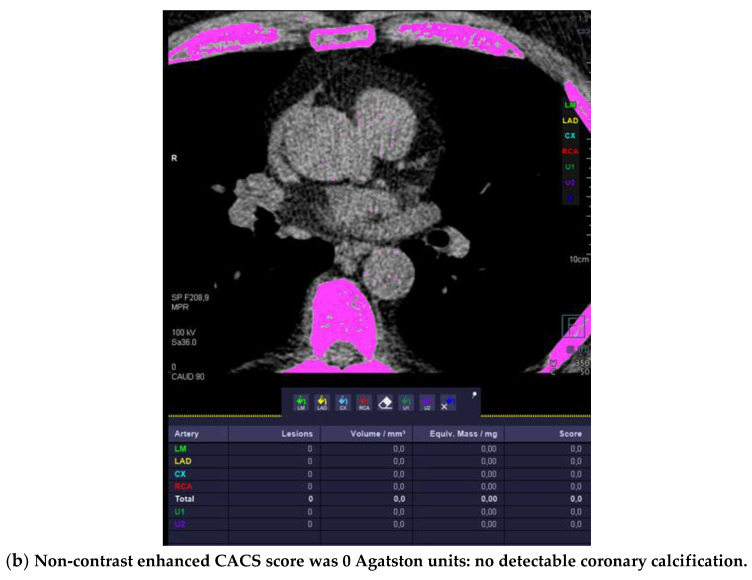
High-risk plaque (HRP) in a patient with CACS 0, a 70 years-old-male patient with ST-depression on ECG-treadmill stress test in V3-6. CT showed 37% diameters stenosis and a high-risk plaque (LAP 44 HU, positive remodeling) (white arrow) in the proximal left anterior descending artery (LAD), which were not detected by CACS (**b**). Diagnosis of non-obstructive CAD by CTA. Distal segment with deep myocardial bridging (MB), explaining stress test findings. (**a**) CTA (left: cMPR and right: 3-DVRT, spider view). LAD = left anterior descending. CX = circumflex artery.

Out of those 103 with CAD and CACS 0, 52 (50.4%) had at least one HRP per patient (Figure 1).

## 4. Discussion

Our data clearly show that in symptomatic high-risk patients, CACS 0 does not rule out the presence of non-obstructive CAD. However, the presence of obstructive CAD (>50% stenosis) is extremely rare (0.61%), allowing for a relatively safe exclusion of obstructive CAD in symptomatic patients with CACS 0. This is of importance in specific clinical settings, such as acute critical care or other in-hospital setting, such as prior to urgent or major surgery in which a non-invasive exclusion of obstructive CAD is required, while certain conditions do not allow for contrast agent injection, or pose the patient at a considerable risk (e.g., severe kidney failure, previous anaphylactic reaction to iodine contrast or others). Especially in patients, in whom a quick exclusion of obstructive CAD is required (e.g., urgent or elective surgery for example for an aortic aneurysm, infective endocarditis, liver or kidney transplantation, or any other major non-cardiac surgeries such as orthopedics), CACS 0 is a relatively reliable tool. 

On the other hand, in a primary care outpatient setting focusing on early detection and prevention of coronary heart disease—the diagnosis of non-obstructive CAD is of greater impact and relevance. Non-obstructive CAD is nowadays regarded as a novel diagnosis for defining primary cardiovascular (CV) prevention strategies according to the AHA/ACC chest pain guideline [14]. For example, a lower c-LDL value should be targeted, especially in the presence of high-risk plaque, in order to prevent adverse outcomes. Moreover, communication with the patient should include discussion of diagnosis, and lifestyle as well as therapeutic interventions for primary cardiovascular prevention discussed. For example, weight loss strategies in case of obesity, smoking cessation, dietary recommendations (e.g., Mediterranean diet, sugar free and low-carb/low saturated fat diet), regular exercise >3–5 times/week at moderate intensity and others, should be enforced in those patients. It is more likely, that patients will adhere to lifestyle interventions with an established diagnosis and being aware of the risk, than without.

Our results are in line with a recent large Danish cohort study [5], showing in 23,759 patients that CACS 0 allowed for a reliable exclusion of major adverse cardiac events (MACE) such as all-cause and CV mortality. If CACS is 0, the risk of MACE is similar to the general population. In contrast to the Danish cohort, our study included the semi-quantitative assessment of “high-risk-plaque” (HRP) features—as a novelty.

HRP are independent risk factors for adverse outcomes, which even outperform CACS and stenosis severity [10], as shown in the prospective randomized SCOT-HEART trial. Other than gender, certain cardiovascular (CV) risk factors influence (smoking and obesity) [15] the increase of the likelihood of HRP. In general, males present more often with HRP than females [16]. Therefore, an individual approach is advisable in order to estimate whether nonobstructive CAD may be present in an individual with CACS 0. In summary, male sex, young age <40 years, diabetes, smoking and obesity certainly are risk factors. Beyond, the combination of plaque features, lesion morphology and stenosis defines ischemia more accurately, than stenosis severity [12,17]. In patients with HRP, CV risk factors should be controlled even more relentlessly, than in those with non-obstructive CV but without HRP. In another multicentric study (CAPIRE) (including patients with coronary calcium), HRP were associated with male sex, older age, diabetes and biomarkers of inflammation such as c-reactive protein and PTX-3 [18].

### Limitations

We did not collect CV outcomes such as MACE, because multiple large cohort studies have already extensively been proven the “powerofCACS0” and its safety in various cohorts [4,5]. Of note, the majority (>95%) of our patients had stable chest pain, and a non-acute clinical presentation. Only a few patients had acute chest pain and borderline or very mild troponin elevation. Therefore, our study does not allow for conclusion on the safety of CACS 0 to rule out obstructive CAD in acute care patients presenting with troponin elevation and/or dynamic. In those patients, the prevalence of obstructive CAD may be considerably higher, and reach values reported by Mortensen et al. (5), or even beyond.

High-risk plaques (HRP) were analyzed semiquantitatively [19], according to established and validated methods [6,7,8,9,10,11]. This approach offers the advantage of an exact visual validation of measurement accuracy, and the exclusion of artifacts, such from motion or ECG-misalignment, which may inhibit—or cause errors—using a fully automated software analysis. However, semiquantitative evaluation is time-consuming and cumbersome in daily clinical practice.

Novel fully automated quantitative AI-based software for plaque analysis [20] is promising for a quicker and a more convenient clinical application.

CT technology does play an important role for the accurate quantification of HRP. By using older CT scanner generation prior to 2011 [21], HRP, often, could not be accurately analyzed. Up to approximately 30% of CT scans were rejected from quantitative cardiac analysis due to artifacts. The introduction of dual-source CT with higher temporal resolution has already markedly improved image quality and the detection of high-risk plaque, allowing for a routine clinical implementation.

Most recent technological developments such as photo-counting CT yield to a further improvement in image quality and higher spatial resolution [22], which is crucial for an accurate quantitative analysis of small lesions such as coronary plaque.

## 5. Conclusions

The prevalence of atherosclerosis in symptomatic patients with CACS 0 is low but not negligible (15.4%), and coronary artery disease is mostly (96.1%) non-obstructive. Non-obstructive CAD is a novel important diagnosis according to the new AHA chest pain guideline 2021 [2] for defining primary prevention strategies [14], such as controlling blood pressure and metabolic risk factors, c-LDL lowering therapy and other lifestyle modifications [23].

Importantly, high-risk plaque prognosticate for further cardiac events [6,7,8,9,10,11], hence improving CV risk stratification and influencing clinical management, such as c-LDL lowering therapy. Lower c-LDL targets should be aimed in patients with high-risk plaque, and lifestyle modifications (such as weight loss, regular exercise and nutrition) emphasized and intensified. Novel data from the large-scale PURE substudy [24] are pinpointing the powerful impact of modifiable CVD risk factors (such as abdominal obesity, poor diet, low strength and others), which were contributing to the majority of total adjusted population-attributable fractions (PAFs) in China and South Asia with 59% and 64%, and even 72% in Brazil [25].

In clinical practice, CACS 0 cannot be used to safely rule out non-obstructive CAD, while obstructive CAD is very unlikely.

If non-obstructive CAD is present, high-risk plaque are common (50.4%), highlighting the clinical relevance of quantitative plaque analysis.

## Figures and Tables

**Table 1:** Study population (*n* = 1709).

**Age (years)**	57.5 ± 16.4
**Females**	667 (39.6%)
**BMI (kg/m^2^)**	26.3 ± 5.2
**Active smoking**	476 (27.8%)
**Arterial hypertension**	683 (39.8%)
**Positive family history**	806 (47.2%)
**Dyslipidemia**	939 (54.9%)
**Diabetes**	242 (14.2%)

**Table 2:** Cardiovascular risk factors in CACS 0 and CACS positive (>1 AU) patients.

	CACS + *n* = 1043	CACS 0 *n* = 666	*p*-Value
**Age (years)**	56.6 ± 17	57.9 ± 15.9	0.706
**Females**	36.9%	43.4%	0.073
**Art hypertension**	47.3%	22.8%	<0.001 *
**Active smoking**	30.1%	21.2%	0.097
**Positive family history**	46.0%	47.8%	0.837
**Dyslipidemia**	58.6%	40.7%	0.998
**Diabetes**	17.4%	5.2%	0.002 *

* significant. AU = Agatston units.

**Table 3:** CTA results in 666 patients with CACS 0.

CTA	CAD RADS	CTA Stenosis	N	N (%)	CAD	HRP in Patients with CAD?
**CAD −**	0	0	562	562 (84.5%)	0	
**CAD +**	1	1–25%	79	103 (15.4%)	99 (15.1%) **non-ob.**	52 (50.4%)
	2	25–49.9%	20			
	3	50–69.9%	1		4 (0.61%) **obstructive**	
	4	70–99%	3			
**excluded**	N *	Nondiagn.	1	1		
N			666	665	665	103

Abbreviations. CAD = coronary artery disease. CTA = computed tomography angiography. HRP = high risk plaque. Non-ob. = non-obstructive. * N = non-diagnostic image quality due to artifacts. This patient (*n* = 1) was excluded from CADRADS and HRP analysis.

## Data Availability

Not applicable.
